# Susceptibility of the *Candida haemulonii* Complex to Echinocandins: Focus on Both Planktonic and Biofilm Life Styles and a Literature Review

**DOI:** 10.3390/jof6040201

**Published:** 2020-10-01

**Authors:** Lívia S. Ramos, Laura N. Silva, Marta H. Branquinha, André L. S. Santos

**Affiliations:** 1 Laboratório de Estudos Avançados de Microrganismos Emergentes e Resistentes (LEAMER), Departamento de Microbiologia Geral, Instituto de Microbiologia Paulo de Góes (IMPG), Universidade Federal do Rio de Janeiro (UFRJ), Rio de Janeiro 21941-901, Brazil; liviaramos2@yahoo.com.br (L.S.R.); lauransilva@gmail.com (L.N.S.); mbranquinha@micro.ufrj.br (M.H.B.); 2 Programa de Pós-Graduação em Bioquímica (PPGBq), Instituto de Química (IQ), Universidade Federal do Rio de Janeiro (UFRJ), Rio de Janeiro 21941-909, Brazil

**Keywords:** *Candida haemulonii* complex, planktonic growth, biofilm formation, echinocandins, caspofungin, micafungin

## Abstract

*Candida haemulonii* complex (*C. haemulonii*, *C. duobushaemulonii* and *C. haemulonii* var. *vulnera*) is well-known for its resistance profile to different available antifungal drugs. Although echinocandins are the most effective class of antifungal compounds against the *C. haemulonii* species complex, clinical isolates resistant to caspofungin, micafungin and anidulafungin have already been reported. In this work, we present a literature review regarding the effects of echinocandins on this emergent fungal complex. Published data has revealed that micafungin and anidulafungin were more effective than caspofungin against the species forming the *C. haemulonii* complex. Subsequently, we investigated the susceptibilities of both planktonic and biofilm forms of 12 Brazilian clinical isolates of the *C. haemulonii* complex towards caspofungin and micafungin (anidulafungin was unavailable). The planktonic cells of all the fungal isolates were susceptible to both of the test echinocandins. Interestingly, echinocandins caused a significant reduction in the biofilm metabolic activity (viability) of almost all fungal isolates (11/12, 91.7%). Generally, the biofilm biomasses were also affected (reduction range 20–60%) upon exposure to caspofungin and micafungin. This is the first report of the anti-biofilm action of echinocandins against the multidrug-resistant opportunistic pathogens comprising the *C. haemulonii* complex, and unveils the therapeutic potential of these compounds.

## 1. Introduction

The members of the *Candida haemulonii* species complex (*C. haemulonii*, *C. duobushaemulonii* and *C. haemulonii* var. *vulnera*) are well-known for their (multi)drug-resistance towards several antifungal agents available in clinical practice. Resistance of the *C. haemulonii* complex to azoles (e.g., fluconazole, itraconazole and voriconazole) and polyenes (e.g., amphotericin B) has been documented extensively [1,2,3,4,5,6,7]. On the other hand, susceptibility to prescribed echinocandins (anidulafungin, caspofungin and micafungin) is commonly observed [7,8,9,10,11], although there have been some reports of clinical isolates being resistant to these compounds [5,12].

Echinocandins are the newest class of antifungal agents to be used in clinical practice, exhibiting fungicidal activity against yeasts as well as having a good safety profile [8]. In this sense, the guidelines of the Centers for Disease Control and Prevention (CDC, USA) strongly recommend that echinocandins should be the first choice for the treatment of candidemia in both neutropenic and non-neutropenic patients [9]. The mechanism of action of the echinocandins involves the noncompetitive inhibition of the enzyme β-(1,3)-d-glucan synthase, which is involved in the synthesis of the polysaccharide glucan, resulting in the loss of cell wall integrity and severe stress in the fungal wall [8].

The three clinically available echinocandins usually exhibit both in vitro and in vivo fungicidal activity against a variety of *Candida* species, including those that are intrinsically resistant to azoles or amphotericin B (e.g., *C. krusei*, *C. glabrata* and *C. lusitaniae*), and also emerging species (e.g., *C. famata* and *C. rugosa*) [10]. Additionally, the antifungal activity of echinocandins against *Candida* biofilms represents an aspect that should be highlighted, since microbial biofilm is considered a resistance structure that precludes efficient antimicrobial treatment [10]. For instance, both caspofungin and micafungin, at concentrations attainable in clinical treatments, were able to kill fungal cells in preformed biofilms of either *C. albicans* or *C. parapsilosis* [11]. Therapeutic concentrations of caspofungin and micafungin were active against the biofilms formed by isolates of *C. albicans* and *C. glabrata* recovered from cases of bloodstream infections, but not against *C. tropicalis*, demonstrating that species-specific differences can influence the outcome [12]. Corroborating these findings, caspofungin was also shown to be effective in the treatment and prevention of *C. albicans* biofilms in an in vivo murine model of central venous catheter-associated candidiasis [13].

Considering the aforementioned aspects, the aim of the present study was to evaluate the antifungal susceptibility of both planktonic- and biofilm-forming cells from 12 Brazilian clinical isolates comprising the *C. haemulonii* complex towards caspofungin and micafungin. Furthermore, we have performed a literature review concerning the susceptibility of the *C. haemulonii* species complex towards echinocandins in order to present a comprehensive summary of this field.

## 2. Materials and Methods

### 2.1. Microorganisms and Growth Conditions

Twelve clinical fungal isolates, previously identified by molecular methods [6], belonging to the *C. haemulonii* species complex were used in the present study: five isolates of *C. haemulonii* (LIP*Ch*2 recovered from the sole of the foot, GenBank accession number KJ476194; LIP*Ch*3 from a toe nail, KJ476195; LIP*Ch*4 from a finger nail, KJ476196; LIP*Ch*7 from a toe nail, KJ476199; LIP*Ch*12 from blood, KJ476204), four isolates of *C. duobushaemulonii* (LIP*Ch*1 from finger nail, KJ476193; LIP*Ch*6 from a toe nail, KJ476198; LIP*Ch*8 from blood, KJ476200 and LIP*Ch*10 from bronchoalveolar lavage, KJ476202) and three isolates of *C. haemulonii* var. *vulnera* (LIP*Ch*5 from a toe nail, KJ476197; LIP*Ch*9 from urine, KJ476201 and LIP*Ch*11 from blood, KJ476203) [6]. In all experiments, Sabouraud dextrose medium was used to culture the fungal isolates at 37 °C for 48 h under constant agitation (200 rpm). Yeasts were counted in a Neubauer chamber.

### 2.2. Determination of Minimal Inhibitory Concentration (MIC)

Antifungal susceptibility testing, using the planktonic cells of *C. haemulonii* species complex, against caspofungin and micafungin (Sigma-Aldrich, St. Louis, MO, USA) was performed according to the broth microdilution technique standardized in the M27-Ed4 protocol [14] and interpreted according to the M27-S3 document published by the Clinical and Laboratory Standards Institute (CLSI) [15]. *C. krusei* (ATCC 6258) and *C. parapsilosis* (ATCC 22019) were used as quality control isolates in each test as directed by the CLSI. The clinical breakpoints to echinocandins are detailed below.

### 2.3. Echinocandins’ Breakpoints

Until now, there have been no established breakpoints for echinocandins (or any other antifungal class) regarding the species belonging to the *C. haemulonii* complex. To overcome this problem, researchers working with this fungal complex, as well as “newly identified” *Candida* species, have generally been using a comparative perspective in order to interpret and discuss antifungal susceptibilities. Results are normally presented as CLSI breakpoints which have been established for the *Candida* genus (CLSI document M27S3 [15]) in order to have a minimum (even if not precise) parameter to interpret this kind of experiment. Alternatively, a possible option is to compare the MIC values of *C. haemulonii* complex with the breakpoints established for non-*albicans Candida* species (e.g., *C. glabrata*, *C. tropicalis*, *C. krusei*, *C. parapsilosis* and *C. guilliermondii*) as recently suggested by the CLSI (document M27S4 [16] and protocol M60 [17]). However, this approach varies depending on the particular *Candida* species, since each presents its own breakpoint for each of the echinocandin drugs used. Moreover, the CDC (USA) recently published on its website (https://www.cdc.gov/fungal/candida-auris/c-auris-antifungal.html) a proposal of echinocandins’ breakpoints for *C. auris*, a phylogenetically related species to the *C. haemulonii* complex, as follows: resistant breakpoint for caspofungin is ≥2 mg/L and for micafungin and anidulafungin, ≥4 mg/L. After contemplating these various viewpoints, we chose to use, herein, the breakpoints available for *Candida* spp. in the CLSI document M27-S3 [15], which considers as susceptible the strains having MIC values ≤ 2 mg/L and non-susceptible those with MIC values > 2 mg/L for the three clinically available echinocandins; a MIC summary table was prepared.

### 2.4. Effects of Echinocandins on the Biofilm Formed by the C. haemulonii Species Complex

Fungal suspensions in Sabouraud broth (200 µL containing 10^6^ yeast cells) were transferred into each well of a flat-bottom 96-well polystyrene microtiter plate and incubated without agitation at 37 °C for 48 h, which has been shown to be the best incubation time for biofilm formation by species belonging to the *C. haemulonii* complex [18]. Afterwards, the biofilm supernatant fluids were carefully removed, washed once with sterile phosphate-buffered saline (PBS; 10 mM NaH_2_PO_4_, 10 mM Na_2_HPO_4_, 150 mM NaCl, pH 7.2) and then 200 µL of Roswell Park Memorial Institute Medium (RPMI) 1640 medium containing different concentrations of echinocandins (range 0.25–8 mg/L) were added to each well. RPMI 1640 medium without echinocandins was used as a positive control and medium-only blanks were used as the negative control. The biofilms were then incubated at 37 °C for an additional 48 h. Afterwards, the supernatant fluids were carefully removed and the wells were washed twice with PBS to remove any non-adherent cells. Finally, two classic biofilm parameters (biomass and metabolic activity/viability) were measured as described below. The results were expressed as percentage of reduction of both viability and biomass. The minimal biofilm eradication concentration (MBEC) was achieved, considering the lowest concentration of each echinocandin capable of causing a 50% reduction in the biofilm viability [19].

#### 2.4.1. Viability Assay

The viability of the fungal cells forming the biofilm was determined using a colorimetric assay that measures the metabolic reduction of 2,3-bis(2-methoxy-4-nitro-5-sulfophenyl)-5-[(phenylamino) carbonyl]-2H-tetrazolium hydroxide (XTT; Sigma-Aldrich) to a water-soluble brown formazan product [20,21]. A XTT/menadione solution was prepared as follows: 2 mg of XTT was dissolved in 10 mL of pre-warmed PBS solution supplemented with 100 μL of a menadione stock solution (made by dissolving 55 mg of menadione in 100 mL of acetone). The XTT/menadione solution (200 μL) was added to all wells containing the biofilms (see Section 2.4 above) and incubated in the dark at 37 °C for 3 h. One hundred microliters of the supernatant from each well were then transferred to a new microplate and the colorimetric readings were measured at 492 nm using a microplate reader (SpectraMax M3; Molecular Devices, Sunnyvale, CA, USA) [21].

#### 2.4.2. Biomass Measurement

Biomass quantification was assessed as described by Peeters et al. [20]. Firstly, biofilms (see Section 2.4 above) were fixed by adding 200 μL of 99% methanol for 15 min. The supernatant was then discarded. Microtiter plates were air-dried for 5 min and then 200 μL of 0.4% crystal violet solution (stock solution diluted in PBS; Sigma-Aldrich) were added to each well and the plates then incubated at room temperature for 20 min. After discarding the crystal violet solution, the wells were washed once with PBS to remove excess stain and the biomass in each well was then decolorized by adding 200 μL of 33% acetic acid for 5 min. One hundred microliters of the acetic acid solution were transferred to a new 96-well plate and the absorbance measured at 590 nm using a microplate reader (SpectraMax M3; Molecular Devices) [21].

### 2.5. Biofilm Architecture: Confocal Laser Scanning Microscopy (CLSM) Assay

Biofilms were formed on a polystyrene surface and treated as described above with different concentrations of micafungin (0.5–2.0 mg/L). Then, the biofilms were stained with Calcofluor white (Sigma-Aldrich) solution (5 µg/mL) for 1 h at room temperature and protected from the light [21,22,23]. Subsequently, the biofilms were washed twice with PBS and covered with *n*-propyl-gallate for observation using a confocal microscope (Leica TCS SP5 with OBS, Berlin, Germany). Fiji ImageJ2 software (UW-Madison LOCI, Madison, WI, USA), was used to obtain three-dimensional (3-D) reconstitutions of the biofilms [21,24]. In this way, image analysis was performed using *z*-series image stacks from five randomly chosen spots on each biofilm [21].

### 2.6. Literature Review

This exercise involved the compilation of available data regarding the susceptibility of the *C. haemulonii* species complex to echinocandins. The literature search was performed on 19 July 2020 using the following four databases: PubMed (https://pubmed.ncbi.nlm.nih.gov), Web of Science (https://webofknowledge.com), Google Scholar (https://scholar.google.com) and Scielo (https://scielo.org/). The term “Candida haemulonii” was added in the category “title/abstract” in the PubMed Advanced Search Builder and in the Web of Science databases, while in Google Scholar the search was conducted in the advanced search area, including the term “Candida haemulonii” and selecting the option “with the exact phrase in the title”; finally, for the Scielo database, we only used the search term “Candida haemulonii” in the general search. Papers available in English and published after the reclassification of the *C. haemulonii* complex by Cendejas-Bueno et al. [5] were selected. Subsequently, the list of results from each database was exported to the EndNote^®^ software (version X1), using the “Output Records” tool in order to eliminate possibly duplicated references by means of the “Find Duplicates” tool. Finally, the papers were individually analyzed in order to select those that described either MIC or geometric-mean (GM)-MIC values of the *C. haemulonii* complex for echinocandins.

### 2.7. Statistics

All experiments were performed in triplicate, in three independent experimental sets. The results were analyzed statistically by the Analysis of Variance One-Way ANOVA (comparisons between three or more groups). All analyzes were performed using the GraphPad Prism5 program. For all analyses, *p* values of 0.05 or less were considered statistically significant.

## 3. Results and Discussion

### 3.1. Susceptibility of Planktonic Cells of the C. haemulonii Species Complex to Echinocandins

According to the breakpoints suggested in the M27S3 document published by CLSI, the planktonic cells of all clinical isolates of the *C. haemulonii* complex tested herein were considered susceptible to echinocandins, with MIC values ranging from 0.125 to 0.5 mg/L for caspofungin and 0.25–0.5 mg/L for micafungin (Table 1). For instance, a recent report described the successful use of caspofungin (MIC of ≤0.125 mg/L) in the treatment of a case of catheter-related candidemia caused by *C. haemulonii* in a pediatric patient in Mexico [25], whose fungal isolate exhibited in vitro high MICs for azoles (fluconazole MIC ≥256 mg/L, posaconazole ≥8 mg/L, itraconazole, ketoconazole and voriconazole ≥16 mg/L) and amphotericin B (MIC 1–2 mg/L). Some years before, a catheter-related candidemia in an adult patient hospitalized for a long period was only resolved when fluconazole treatment was replaced by caspofungin [4].

In general, echinocandins are highly active in vitro against species comprising the *C. haemulonii* complex [7,26,27,28,29], but the existence of isolates resistant to this class of antifungals has already been reported [4,5,30]. Herein, we conducted a careful review of the literature regarding the susceptibility of the *C. haemulonii* species complex to the three clinically available echinocandins, including only papers published after the species reclassification and the creation of the *C. haemulonii* complex [5]. Using the keyword “Candida haemulonii” in the search section, 148, 63, 46 and 5 publications were located from the Web of Science, PubMed, Google Scholar and Scielo databases, respectively (Table 2). However, only a small fraction of these published papers (varying from 12.2%–28.3%) cited the in vitro susceptibility profile of the *C. haemulonii* species complex against echinocandins. In this sense, we recovered a total of 21 distinct papers that fitted our established criteria and, for these reasons, they were selected for data extraction as follows: 5 (23.8%) papers studied the three members forming the *C. haemulonii* complex, 6 (28.6%) studied only two species (*C. haemulonii* and *C. duobushaemulonii*) and 10 (47.6%) studied only one species (*C. haemulonii*, *n* = 6, *C. duobushaemulonii*, *n* = 3, *C. haemulonii* var. *vulnera*, *n* = 1). Furthermore, 13 (61.9%) papers detailed the MIC value for each isolate investigated, while the remaining studies (*n* = 8; 38.1%) only presented the geometric mean (GM)-MIC and/or the range of MIC values for the fungal isolates against the test echinocandins. Finally, 12 (57.1%) papers tested the three echinocandins, 5 (23.8%) used two and 4 (19.1%) tested only one echinocandin, with caspofungin being the most frequently evaluated.

The results emanating from this literature review revealed that micafungin and anidulafungin appeared to be more effective than caspofungin against the three species forming the *C. haemulonii* complex (Table 3) [5,7,25,29,31,32,33,34,35,36,37,38,39,40,41,42,43,44,45,46,47]. In this respect, 89.8% of the isolates of *C. haemulonii* exhibited susceptibility to caspofungin, while 96.3% and 98.4% were susceptible to micafungin and anidulafungin, respectively. Regarding *C. duboushaemulonii*, 95.5% of the isolates were susceptible to caspofungin, 99.1% to anidulafungin and 100.0% to micafungin. Finally, considering the clinical isolates of *C. haemulonii* var. *vulnera*, 85.0% were susceptible to caspofungin, 91.7% to micafungin and 97.1% to anidulafungin. Indeed, the MIC frequency distribution demonstrated that the modal MIC of echinocandins against the *C. haemulonii* complex was ≤0.12 mg/L in almost all cases (Table 4).

Comparing the GM-MIC values of our clinical isolates (Table 1) with those compiled from the literature reports (for these comparisons, we used the arithmetic mean of the GM-MIC values of the selected works, as summarized in Table 5), we observed that the GM-MIC values of caspofungin for our isolates of *C. haemulonii*, *C. duobushaemulonii* and *C. haemulonii* var. *vulnera* were higher than those reported in the literature (0.33 mg/L versus 0.18 mg/L for *C. haemulonii*, 0.18 mg/L versus 0.11 mg/L, for *C. duobushaemulonii* and 0.32 mg/L versus 0.21 mg/L for *C. haemulonii* var. *vulnera*). Similarly, GM-MIC values for micafungin calculated from the literature reports were lower than ours (0.18 mg/L versus 0.33 mg/L for *C. haemulonii*, 0.17 mg/L versus 0.30 mg/L for *C. duobushaemulonii*, and 0.13 mg/L versus 0.25 mg/L for *C. haemulonii* var. *vulnera*). Finally, based on the analysis of the literature data, anidulafungin also produced low GM-MIC values for the three fungal species of the *C. haemulonii* complex (GM-MICs of 0.16, 0.32 and 0.06 mg/L for *C. haemulonii*, *C. duobuhaemulonii* and *C. haemulonii* var. *vulnera*, respectively).

In summary, the majority of literature reported GM-MIC concentration values of <0.5 mg/L for the three echinocandins against the *C. haemulonii* species complex. Nevertheless, two works warranted specific attention: Cendejas-Bueno et al. [5], in which the GM-MIC values for caspofungin for the three members of the *C. haemulonii* complex were disproportionately high in comparison to our present results and those given in the other literature publications; and Isla et al. [36], in which the GM-MIC value obtained for caspofungin against the *C. duobushaemulonii* isolates was considerably higher (Table 5). A possible explanation for the high MIC values found in the aforementioned papers is the possible occurrence of paradoxical growth effect (also known as the Eagle effect), that is characterized by reduced activity of the antifungal agents at high concentrations. In fact, Cendejas-Bueno et al. [5] stressed this discussion in their study, but in a superficial way. A recent study conducted with 106 clinical isolates of *C. auris* demonstrated that the vast majority of isolates were susceptible to the echinocandins; however, they exhibited different intensities of paradoxical growth effect in the presence of caspofungin, whilst four isolates were resistant to echinocandins and had a mutation in hot spot region 1 of the *FKS* gene [48]. Interestingly, those isolates presenting paradoxical growth effect were susceptible to caspofungin at doses used in human treatment, while those with *FKS1* mutation were still resistant in a murine model of invasive candidiasis, demonstrating that only the isolates with the mutations display in vivo echinocandin resistance [48].

### 3.2. Effects of Echinocandins on the Biofilm Formed by C. haemulonii Species Complex

**Figure 1:**
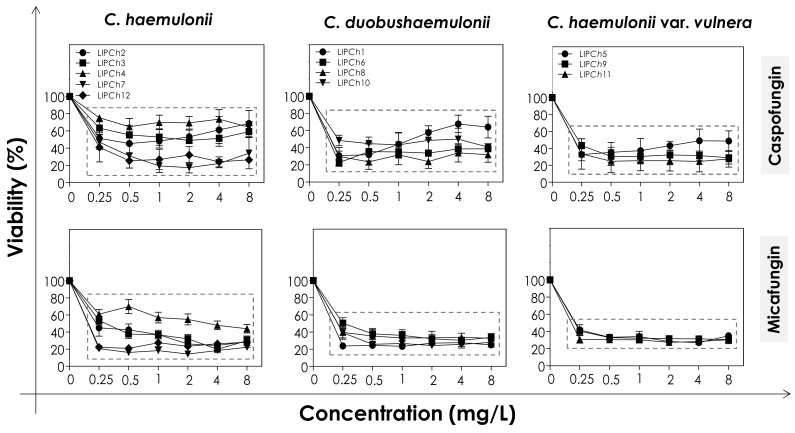
Cell viability of biofilms formed by clinical isolates comprising the *C. haemulonii* complex exposed to different concentrations of echinocandins (caspofungin and micafungin). The results were assessed spectroscopically (492 nm) by XTT reduction and expressed as the mean of metabolic activity percentages compared to untreated biofilms (control), which correspond to 100%. The graphs exhibit the mean ± standard deviation of three independent experiments. The dashed boxes represent the concentrations of echinocandins that caused statistically significant reduction of cell viability in relation to the respective control (*p* < 0.05; One-way ANOVA analysis of variance, Dunnett’s multiple comparison test).

**Figure 2:**
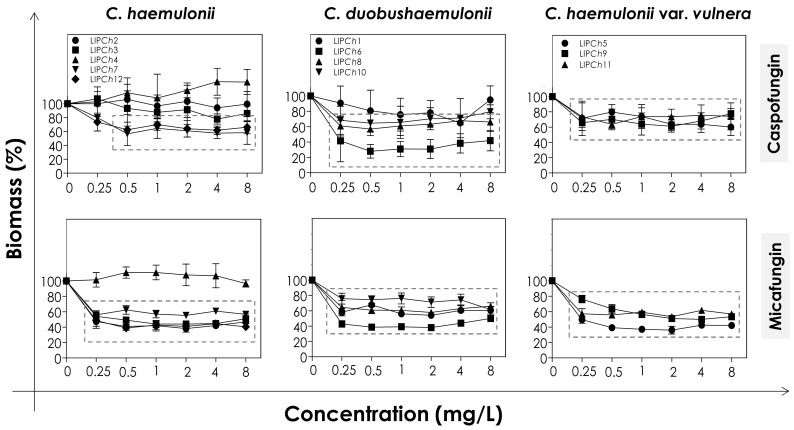
Biomass of biofilms formed by clinical isolates comprising the *C. haemulonii* species complex exposed to different concentrations of echinocandins (caspofungin and micafungin). The amount of crystal violet incorporated by the cells was assessed spectroscopically (absorbance at 590 nm) and the results expressed as the mean of biomass percentages compared to untreated biofilms (control), which correspond to 100%. The graphs show the mean ± standard deviation of three independent experiments. The dashed boxes represent the concentrations of echinocandins that caused a statistically significant reduction in biomass in relation to the respective control (*p* < 0.05; One-way ANOVA analysis of variance, Dunnett’s multiple comparison test).

In order to evaluate the effects of echinocandins (caspofungin and micafungin) on the viability and biomass of the biofilms formed by the clinical isolates of the *C. haemulonii* complex, the mature biofilms were firstly incubated with different concentrations of the antifungals and then analyzed. The metabolic activity of viable fungal cells was assessed by their ability to reduce XTT to formazan, whilst the decrease in biofilm biomass was measured spectroscopically by looking at the incorporation of crystal violet into methanol-fixed, non-viable cells (Figure 1 and Figure 2). In general, the test echinocandins were found to be more efficient at reducing cell viability than decreasing the biomass of the *C. haemulonii* complex biofilms.

The decrease of both viability and biomass parameters by caspofungin was isolate-dependent. At the lowest concentration used (0.25 mg/L) this echinocandin caused a statistically significant reduction in the viability of all of the fungal cells tested (*p* < 0.05; One-way ANOVA analysis of variance, Dunnett’s multiple comparison test), varying from 30–80% among the different isolates (Figure 1). However, caspofungin was unable to reduce the biomass of some of the *C. haemulonii* isolates (LIP*Ch*2, LIP*Ch*3 and LIP*Ch*4) even at the highest concentration used. Nevertheless, for the remaining fungal isolates the drug caused a biomass reduction of up to 60% (mainly against the *C. duobushaemulonii* isolates) (Figure 2). The isolates LIP*Ch*2 (*C. haemulonii*), LIP*Ch*1 (*C. duobushaemulonii*) and LIP*Ch*5 (*C. haemulonii* var. *vulnera*) were less susceptible to caspofungin at the higher concentrations (Figure 1).

Micafungin proved to be more effective than caspofungin at disturbing both biofilm viability and biomass. A decrease in biofilm viability of up to 60% was seen among most of the clinical isolates, especially against *C. duobushaemulonii* and *C. haemulonii* var. *vulnera* (Figure 1). Unlike caspofungin, micafungin showed a decrease of up to 60% on the biofilm biomass of *C. haemulonii* isolates, with the exception of isolate LIP*Ch*4, which forms a very dense and robust biofilm (Figure 2). For the *C. duobushaemulonii* and *C. haemulonii* var. *vulnera* isolates, micafungin reduced biomass in the range 20–60% (Figure 2). In summary, the lowest concentration of micafungin used was able to significantly reduce the cell viability and the biomasses of biofilms formed by all of the test isolates, expect for the biomass of one isolate.

The determination of MBEC, which was defined as the lowest antifungal concentration able to reduce the biofilm viability in 50% [19], revealed that the biofilms of all isolates remained susceptible to echinocandins, with the exception of the isolate LIP*Ch*4 of *C. haemulonii* (Table 6). This fact could be explained by the ability of the isolate LIP*Ch*4 to form very robust biofilm on polystyrene in comparison with the other isolates [18,21], hampering the action of echinocandins due to the high amount of fungal cells-forming the biofilm architecture as well as due to the high production of extracellular matrix that can block the antifungal penetration into the biofilm structure.

**Figure 3:**
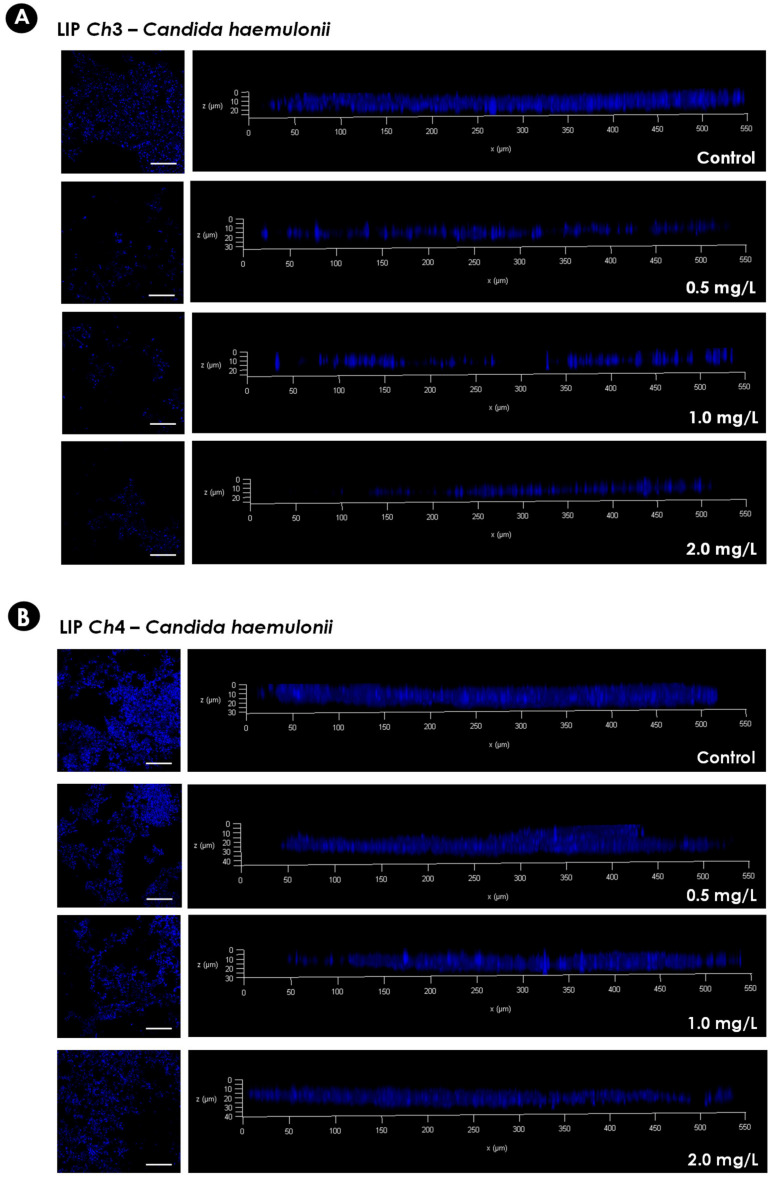
Representative confocal laser scanning microscopy (CLSM) images of the biofilms formed by *C. haemulonii* on a polystyrene surface. Yeasts (200 μL containing 10^6^ cells) were placed to interact with the polystyrene for 48 h at 37 °C. Subsequently, the supernatant fluids were removed and washed with PBS, and 200 µL of RPMI 1640 medium containing different concentrations of micafungin were added. The biofilms were incubated at 37 °C for an additional 48 h. Afterwards, the supernatant fluids were carefully removed again, and the wells were washed twice with PBS to remove non-adherent cells. Finally, the biofilms were stained with Calcofluor white in order to evidence the fungal biomass. The panels on the left represent the top view images of the fungal biofilms visualized by Confocal Laser Scanning Microscopy (CLSM) (bars represent 5 µm). The graphs on the right represent the three-dimensional reconstruction of the biofilms formed. The isolate LIP*Ch*3 of *C. haemulonii* (**A**) was chosen to represent susceptible biofilms, while the isolate LIP*Ch*4 of *C. haemulonii* (**B**) represents resistant biofilms.

As micafungin was more active than caspofungin against the mature biofilms formed by the *C. haemulonii* species complex it was chosen for further studies. In order to verify the 3-D organization of the biofilms following exposure to micafungin two isolates of *C. haemulonii* were selected: LIP*Ch*3, to represent the isolates having susceptible biofilms, and LIP*Ch*4, to represent isolates forming resistant biofilms. CLSM analysis was conducted using Calcofluor white, which binds to the chitin in the fungal cell wall, to evidence the biofilm biomass. The CLSM analysis corroborated the results observed by crystal violet approach, with the lowest antifungal concentration used causing a drastic reduction in the biofilm biomass of LIP*Ch*3, whilst even the highest concentration of micafungin failed to affect the biofilm formed by LIP*Ch*4 (Figure 3).

Until now, no information has been available in the literature regarding the activity of conventional antifungal agents against the biofilm formed by the *C. haemulonii* species complex. A recent study conducted with *C. auris*, which belongs to the *C. haemulonii* clade, showed that, despite the susceptibility of planktonic cells to echinocandins and amphotericin B, the biofilms were not vulnerable, exhibiting MBECs which were 512-fold higher than their planktonic MIC counterparts [19]. Actually, the biofilm formed by *C. auris* is not as robust as those arising from *C. albicans* and *C. glabrata*, but its tolerance to the major classes of antifungal agents is notable, especially for amphotericin B and micafungin, which are the recommended antifungal therapeutics for infections caused by *C. albicans* biofilms [49]. The antifungal tolerance of the *C. auris* biofilm has been shown to be phase-dependent, with the mature biofilms resistant to the three available antifungal drug classes [50]. On the other hand, micafungin has been shown to be effective against both planktonic and biofilm-forming *C. albicans* cells, while its effectiveness against *C. parapsilosis* was considered to be moderate [51]. Additionally, micafungin concentrations >2 mg/L prevented the regrowth of *Candida* biofilm cells [51]. Regarding the *C. parapsilosis* complex, caspofungin was more active against biofilms of *C. orthopsilosis* than *C. parapsilosis* sensu strictu, with 20% and 86% of isolates resistant to this antifungal, respectively, suggesting that a treatment of catheter-related candidemia caused by *C. orthopsilosis* with caspofungin would be more effective than against *C. parapsilosis* sensu strictu [52]. A study, conducted with five different *Candida* species recovered from cases of bloodstream infections demonstrated both species-specific and drug-specific differences in *Candida* biofilms regarding their susceptibility to echinocandins [53]. In this sense, while *C. albicans* and *C. krusei* biofilms were susceptible to the three clinically available echinocandins, *C. lusitaniae*, *C. guilliermondii* and *C. parapsilosis* were quite resistant to them [53]. In addition, micafungin seemed to be the most effective echinocandin against *C. parapsilosis* biofilms, presenting lower MBECs against this *Candida* species in comparison to caspofungin and anidulafungin [53]. These observations reinforce the need to determine the correct identification of the actual fungal species causing the candidiasis infection and, further, to assess its antifungal susceptibility profile against both planktonic and biofilm-forming cells in order to choose the best therapeutic option for each case.

Furthermore, we observed that one isolate of each species forming the *C. haemulonii* complex showed a smaller reduction in cell viability when incubated in the presence of higher concentrations of the echinocandins. This phenomenon is called paradoxical growth, and it corresponds to the decreased sensitivity to echinocandins in the presence of concentrations higher than the MIC values. To date, the evidence strongly suggests that this paradoxical effect is more commonly associated with caspofungin than either micafungin or anidulafungin [54]. This effect has already been documented for biofilms formed by other *Candida* species, such as *C. albicans* [53,55], *C. parapsilosis* [53], *C. tropicalis* [55] and *C. dubliniensis* [56].

To finalize, we recognize some of the limitations associated with the present study, such as the limited number of isolates used and the exclusion of anidulafungin. The experiments were conducted with only 12 clinical isolates of the *C. haemulonii* complex due to the difficulties in obtaining more isolates, since it is quite a rare fungal complex. Additionally, we tested only two of the three echinocandins currently in clinical use, and this was because at the time the experiments were conducted anidulafungin was not available for scientific research purposes.

## 4. Conclusions

In addition to their own clinical conditions, hospitalized patients are at constant risk of acquiring contagions associated with the hospital environment. Biofilm-related *Candida* infections represent an important and worrisome threat to these patients, and there is a limited number of available antifungal agents of sufficient potency to break down these highly resistant structures. In this sense, echinocandins are considered highly active against various *Candida* species and the results presented herein reinforce the potential of echinocandins to treat biofilm-related infections caused by the emergent and multidrug-resistant species comprising the *C. haemulonii* complex.

## Figures and Tables

**Table 1:** MIC values of echinocandins against the *C. haemulonii* species complex studied herein.

Fungal Species	MIC (mg/L)	
Isolates	Caspofungin ^b^	Micafungin
***C. haemulonii***		
LIP*Ch*2	0.5	0.25
LIP*Ch*3	0.5	0.5
LIP*Ch*4	0.5	0.5
LIP*Ch*7	0.25	0.25
LIP*Ch*12	0.125	0.25
GM-MIC ^a^	**0.33**	**0.33**
Arithmetic mean	**0.37**	**0.35**
***C. duobushaemulonii***		
LIP*Ch*1	0.125	0.25
LIP*Ch*6	0.25	0.5
LIP*Ch*8	0.125	0.25
LIP*Ch*10	0.25	0.25
GM-MIC	**0.18**	**0.30**
Arithmetic mean	**0.19**	**0.31**
***C. haemulonii* var. *vulnera***		
LIP*Ch*5	0.25	0.25
LIP*Ch*9	0.25	0.25
LIP*Ch*11	0.5	0.25
GM-MIC	**0.32**	**0.25**
Arithmetic mean	**0.33**	**0.25**
Overall GM-MIC	**0.26**	**0.30**
Overall arithmetic mean	**0.30**	**0.31**

^a^ GM-MIC, geometric mean-minimal inhibitory concentration. ^b^ Similar results were reported in our previously published paper [6]. Journal of Antimicrobial Chemotherapy (JAC) provided the permission to reproduce this set of results.

**Table 2:** Number of publications retrieved from database searches using the term “Candida haemulonii”.

Database	Total Number of Papers	Number of Selected Papers *	References of the Selected Papers *
Web of Science	148	18	[5,7,25,29,31,32,33,34,35,36,37,38,39,40,41,42,43,44]
PubMed	63	16	[5,7,25,29,31,32,33,34,35,39,40,41,43,44,45,46]
Google Scholar	46	13	[5,7,25,29,31,32,33,35,36,40,44,46,47]
Scielo	5	1	[31]

The searches were conducted in PubMed (https://pubmed.ncbi.nlm.nih.gov), Web of Science (https://webofknowledge.com), Google Scholar (https://scholar.google.com/) and Scielo (https://scielo.org) on 19 July 2020. The term “Candida haemulonii” was added in the category “title/abstract” in the PubMed Advanced Search Builder and Web of Science; in Google Scholar the search was conducted in the advanced search area, including the term “Candida haemulonii” and selecting the option “with the exact phrase in the title”; in Scielo, we only searched for the term “Candida haemulonii” in the general search. Papers published after the reclassification of the *C. haemulonii* complex were included [5]. * Papers that evaluated the susceptibility of isolates of the *C. haemulonii* species complex to echinocandins.

**Table 3:** Literature compilation regarding the distribution (%) of the susceptible (S) and non-susceptible (NS) isolates belonging to the *C. haemulonii* complex against echinocandins described in published papers available until 19 July 2020.

Fungal Species	Susceptibility Profile (%) *					
	Caspofungin		Micafungin		Anidulafungin	
	S	NS	S	NS	S	NS
*C. haemulonii*	89.8	10.2	96.3	3.7	98.4	1.6
	***n* = 157**		***n* = 136**		***n* = 185**	
*C. duobushaemulonii*	95.5	4.5	100	0	99.1	0.9
	***n* = 111**		***n* = 105**		***n* = 110**	
*C. haemulonii* var. *vulnera*	85.0	15.0	91.7	8.3	97.1	2.9
	***n* = 20**		***n* = 12**		***n* = 35**	

* Antifungal susceptibility testing was interpreted according to the document M27-S3 published by CLSI; *n*, number of fungal isolates; the references used to construct this table were [5,7,25,29,31,32,33,34,35,36,37,38,39,40,41,42,43,44,45,46,47].

**Table 4:** MIC distribution of *C. haemulonii* complex isolates obtained from the literature review against the three echinocandins.

Drug ^a^ *Species*	MIC (mg/L)													MIC_50_^b^	MIC_90_^c^
	≤0.015	0.03	0.06	0.12	0.25	0.5	1	2	4	8	16	>16	Range		
**CAS**															
*Ch*		19	17	14	12	6	1			1	1	14	0.03–>16	0.12	>16
*Cd*	3	14	18	20	9	4	1			1	1	3	≤0.015–>16	0.12	0.5
*Chv*				2	5	4						3	0.12–>16	0.25	>16
**MCF**															
*Ch*	8	12	28	8	4	1						4	≤0.015–>16	0.06	0.25
*Cd*	2	12	36	12	3	1							0.06–0.5	0.06	0.12
*Chv*			1	4								1	0.06–>16	0.12	0.12
**ANF**															
*Ch*	27	14	19	10	3	1			1			2	≤0.015–>16	0.03	0.12
*Cd*	11	8	17	16	15	4	3	1	1				≤0.015–4	0.12	0.5
*Chv*	8	1	4									1	≤0.015–>16	≤0.015	0.06

^a^ CAS, caspofungin; MCF, micafungin; ANF, anidulafungin; ^b^ MIC_50_, MIC at which 50% of isolates were inhibited; ^c^ MIC_90_, MIC at which 90% of isolates were inhibited; Modal MICs are indicated with underlined numbers; MIC values of <0.03 were allocated as ≤0.015; Clinical and Laboratory Standards Institute (CLSI) document M27S3 suggests the following breakpoints for echinocandins against *Candida* spp.: susceptible ≤ 2 mg/L and non-susceptible > 2 mg/L; the references used to construct this table were [5,29,31,34,35,38,39,40,41,42,44,46,47].

**Table 5:** Literature review on the antifungal susceptibility of different isolates of the *C. haemulonii* complex to echinocandins.

Reference Number	Fungal Species (Number of Isolates)	GM-MIC (Range) *		
		Caspofungin	Micafungin	Anidulafungin
[5] ^•^	*Ch* (*n* = 19)	11.10 ^(#)^ (0.25–>16)	0.17 ^(#)^ (<0.03–>16)	0.06 ^(#)^ (<0.03–>16)
	*Cd* (*n* = 7)	5.38 ^(#)^ (0.5–>16)	0.06 (0.06–0.12)	0.08 ^(#)^ (<0.03–4)
	*Chv* (*n* = 4)	11.31 ^(#)^ (0.5–16)	0.40 ^(#)^ (0.06–>16)	0.20 ^(#)^ (<0.03–>16)
[29] ^•^	*Ch* (*n* = 14)	0.12 (0.125–0.5)	-	0.015 (0.015–0.015)
	*Cd* (*n* = 9)	0.22 ^(#)^ (0.06–16)	-	0.06 (0.015–0.5)
	*Chv* (*n* = 8)	0.26 (0.125–0.5)	-	0.016 (0.015–0.03)
[33] ^•^	*Ch* (*n* = 6)/*Chv* (*n* = 1)	0.18 (0.06–1)	0.27 (0.125–1)	0.45 (0.25–1)
	*Cd* (*n* = 8)	0.13 (0.06–0.25)	0.38 (0.125–1)	0.54 (0.5–1)
[7] ^•^	*Ch* (*n* = 26)	ND (0.03–0.5)	ND (0.06–0.5)	ND (0.015–0.5)
	*Cd* (*n* = 5)	ND (0.06–0.12)	ND (0.06–0.12)	ND (0.06–0.25)
[35] ◦	*Ch* (*n* = 3)	0.5 (0.5–0.5)	0.19 (0.12–0.5)	0.03 (0.03–0.03)
[39] ◦	*Cd* (*n* = 2)	-	0.12 (0.06–0.25)	0.04 (0.03–0.06)
[40] ◦	*Ch* (*n* = 3)	0.10 (0.06–0.125)	0.20 (0.125–0.25)	-
[44] ◦	*Ch* (*n* = 38)	0.06 ^(#)^ (0.03–16)	0.04 (<0.08–0.12)	0.05 (0.03–0.25)
	*Cd* (*n* = 55)	0.07 (0.016–0.5)	0.06 (0.016–0.25)	0.13 (0.016–2)
[32] ^•^	*Ch* (*n* = 7)	0.19 (0.06–1)	0.28 (0.125–1)	0.44 (0.25–1)
	*Cd* (*n* = 5)	0.14 (0.06–0.25)	0.35 (0.125–1)	0.56 (0.5–1)
[45] ^•^	*Ch* (*n* = 21)	-	-	0.10 (0.06–0.25)
	*Cd* (*n* = 13)	-	-	0.10 (0.03–0.5)
	*Chv* (*n* = 15)	-	-	0.13 (0.03–0.25)
[43] ^•^	*Ch* (*n* = 32)	0.104 (ND)	0.106 (ND)	0.103 (ND)
[36] ^•^	*Ch* (*n* = 16)	0.13^(#)^ (0.015–8)	0.11^(#)^ (0.03–8)	0.09 (0.015–0.5)
	*Cd* (*n* = 3)	5.03^(#)^ (1–16)	0.06 (0.015–0.06)	0.79 (0.5–2)
	*Chv* (*n* = 5)	0.12 (0.06–0.25)	0.14 (0.12–0.25)	0.05 (0.015–0.12)
[42] ◦	*Ch* (*n* = 4)	0.06 (0.03–0.12)	-	-
[47] ◦	*Chv* (*n* = 2)	0.25 (0.25–0.25)	0.12 (0.12–0.12)	0.06 (0.06–0.06)
Arithmetic mean of overall GM-MIC, except for the resistant strains^(#)^				
	*Ch*	0.18 ± 0.15	0.18 ± 0.09	0.16 ± 0.18
	*Cd*	0.11 ± 0.04	0.17 ± 0.15	0.32 ± 0.30
	*Chv*	0.21 ± 0.08	0.13 ± 0.01	0.06 ± 0.05

* GM-MIC, geometric mean of the minimal inhibitory concentrations expressed in mg/L; ^•^ Values of GM-MIC obtained directly from the papers; ◦ Values of GM-MIC calculated by us from the MIC values for each isolate mentioned in the articles; *Ch*, *C. haemulonii*; *Cd*, *C. duobushaemulonii*; *Chv*, *C. haemulonii* var. *vulnera*; *n*, number of isolates studied; arithmetic mean of overall GM-MIC calculated from the GM-MIC of the different papers; ND, not determined; -, no isolates were tested.

**Table 6:** Minimal biofilm eradication concentration (MBEC) to echinocandins against *C. haemulonii* complex.

Echinocandins	MBEC (mg/L)											
	*C. haemulonii Isolates*					*C. duobushaemulonii Isolates*				*C. haemulonii* var. *vulnera Isolates*		
	*Ch*2	*Ch*3	*Ch*4	*Ch*7	*Ch*12	*Ch*1	*Ch*6	*Ch*8	*Ch*10	*Ch*5	*Ch*9	*Ch*11
Caspofungin	0.5	2	˃8	0.25	0.25	˂0.25	˂0.25	˂0.25	0.25	0.25	0.25	0.25
Micafungin	0.25	0.5	8	˂0.25	˂0.25	˂0.25	0.5	0.25	0.25	0.25	˂0.25	˂0.25
